# Supplementary material for: Dynamic epigenetic age mosaicism in the human atherosclerotic artery
Source: PLoS One. 2022 Jun 3;17(6):e0269501. doi: 10.1371/journal.pone.0269501 (PMC9165801; doi:10.1371/journal.pone.0269501)
Supplement: S1 Fig — Differential methylation was significant at genome-wide level (p<10−7, Bonferroni correction; see Table 1 for details). Other associations were significant at nominal significance level (p<0.05; see S1 Table for details). (PPTX) [file pone.0269501.s001.pptx]

## Slide 1
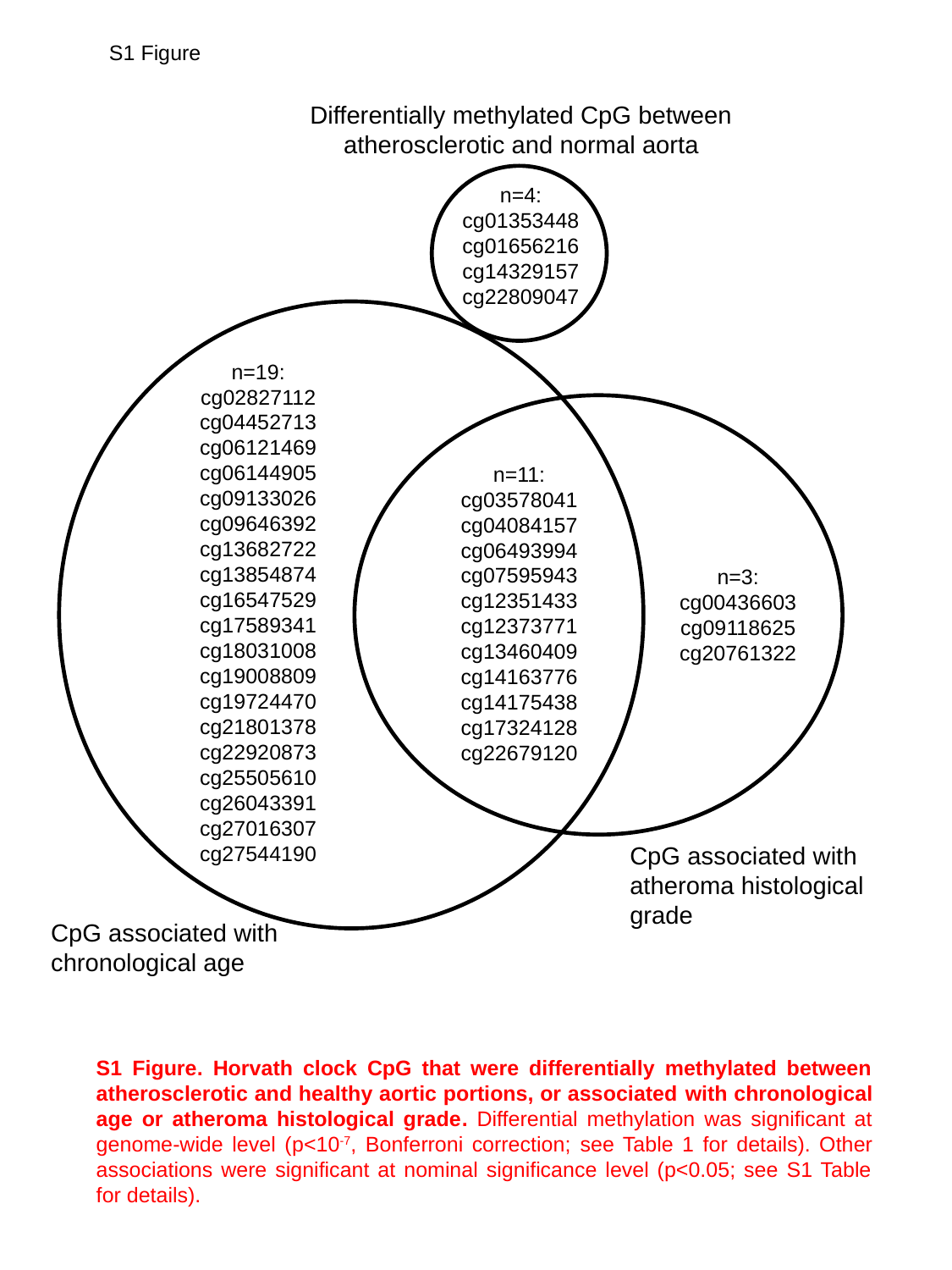

S1 Figure
Differentially methylated CpG between
atherosclerotic and normal aorta
n=4:
cg01353448
cg01656216
cg14329157
cg22809047
n=19:
cg02827112
cg04452713
cg06121469
cg06144905
cg09133026
cg09646392
cg13682722
cg13854874
cg16547529
cg17589341
cg18031008
cg19008809
cg19724470
cg21801378
cg22920873
cg25505610
cg26043391
cg27016307
cg27544190
n=11:
cg03578041
cg04084157
cg06493994
cg07595943
cg12351433
cg12373771
cg13460409
cg14163776
cg14175438
cg17324128
cg22679120
n=3:
cg00436603
cg09118625
cg20761322
CpG associated with
atheroma histological
grade
CpG associated with
chronological age
S1 Figure. Horvath clock CpG that were differentially methylated between atherosclerotic and healthy aortic portions, or associated with chronological age or atheroma histological grade. Differential methylation was significant at genome-wide level (p<10-7, Bonferroni correction; see Table 1 for details). Other associations were significant at nominal significance level (p<0.05; see S1 Table for details).
