# Supplementary material for: Dynamic epigenetic age mosaicism in the human atherosclerotic artery
Source: PLoS One. 2022 Jun 3;17(6):e0269501. doi: 10.1371/journal.pone.0269501 (PMC9165801; doi:10.1371/journal.pone.0269501)
Supplement: S1 Table — (DOCX) [file pone.0269501.s002.docx]

**S1 Table.** Patient information.

| Aortas, n=15 | |
| --- | --- |
| Female (%)  Male (%) | 4 (26.7)  11 (73.3) |
| Age (y±SD) | 64.9±10.6 |
| Histological grade (%):  III  IV  V  VII | 3 (20.0)  2 (13.4)  1 (6.7)  9 (60.0) |
| *Post mortem* time at sample collection (d±SD) | 14.5±5.1 |
| Carotid artery plaques, n=38 | |
| Asymptomatic:  Female (%)  Male (%)  Age (y±SD) | 19  6 (31.6)  13 (68.4)  67.9±5.4 |
| Symptomatic:  Female (%)  Male (%)  Age (y±SD)  Symptom-to-endarterectomy time (d±SD) | 19  4 (21.1)  15 (78.9)  74.5±8.4  21.9±12.8 |
